# Fulminant anaplastic large cell lymphoma (ALCL) concomitant with primary cytomegalovirus (CMV) infection, and human herpes virus 8 (HHV-8) infection together with Epstein-Barr-virus (EBV) reactivation in a patient with asymptomatic HIV-infection

**DOI:** 10.1186/s13027-016-0094-5

**Published:** 2016-08-22

**Authors:** Sven Grützmeier, Anna Porwit, Corinna Schmitt, Eric Sandström, Börje Åkerlund, Ingemar Ernberg

**Affiliations:** 1 Department of Microbiology, Tumor and Cell Biology (MTC), Karolinska Institute, Box 280, Stockholm, SE- 17177 Sweden; 2 Department of Infectious diseases/Venhälsan, Stockholm South General Hospital, Sjukhusbacken 14, SE-11883 Stockholm, Sweden; 3 Department of Oncology/Pathology, Karolinska Institutet, SE- 17177 Stockholm, Sweden; 4 Present address: Department of Laboratory Hematology, Laboratory Medicine Program, University Health Network, Toronto, ON Canada; 5 Institute of Virology, Hannover Medical School, Hannover, Germany; 6 Department of Infectious Diseases, Karolinska University Hospital, Huddinge, Sweden

**Keywords:** Primary CMV-infection, EBV latency, HHV-8 infection, Malignant lymphoma, ALCL, CMV-DNA, EBV-DNA, HHV-8 DNA, Unprotected sex

## Abstract

**Background:**

Most malignant lymphomas in HIV-patients are caused by reactivation of EBV-infection. Some lymphomas have a very rapid fulminant course. HHV-8 has also been reported to be a cause of lymphoma. The role of CMV in the development of lymphoma is not clear, though both CMV and HHV-8 have been reported in tissues adjacent to the tumour in Burkitt lymphoma patients. Here we present a patient with asymptomatic HIV infection, that contracted a primary cytomegalovirus (CMV) infection and human herpes virus 8 (HHV-8) infection. Three weeks before onset of symptoms the patient had unprotected sex which could be possible source of his CMV and also HHV-8 infection He deteriorated rapidly and died with a generalized anaplastic large cell lymphoma (ALCL).

**Methods:**

A Caucasian homosexual male with asymptomatic human immunodeficiency virus (HIV) infection contracted a primary cytomegalovirus (CMV) infection and human herpes virus 8 (HHV-8) infection. He deteriorated rapidly and died with a generalized anaplastic large cell lymphoma (ALCL). Clinical and laboratory records were compiled. Immunohistochemistry was performed on lymphoid tissues, a liver biopsy, a bone marrow aspirate and the spleen during the illness and at autopsy. Serology and PCR for HIV, CMV, EBV, HHV-1–3 and 6–8 was performed on blood drawn during the course of disease.

**Results:**

The patient presented with an acute primary CMV infection. Biopsies taken 2 weeks before death showed a small focus of ALCL in one lymph node of the neck. Autopsy demonstrated a massive infiltration of ALCL in lymph nodes, liver, spleen and bone marrow. Blood samples confirmed primary CMV- infection, a HHV-8 infection together with reactivation of Epstein- Barr-virus (EBV).

**Conclusion:**

Primary CMV-infection and concomitant HHV-8 infection correlated with reactivation of EBV. We propose that these two viruses influenced the development and progression of the lymphoma. Quantitative PCR blood analysis for EBV, CMV and HHV-8 could be valuable in diagnosis and treatment of this type of very rapidly developing lymphoma. It is also a reminder of the importance of prevention and prophylaxis of several infections by having protected sex.

## Background

Cytomegalovirus (CMV) often causes lethal complications in patients with advanced HIV-infection [1, 2]. As the majority of HIV-infected patients are CMV-seropositive at the time of seroconversion little is known about primary CMV infection in HIV-patients with or without severe immunodeficiency. Recently two case reports have been published [3, 4]. The first case was a pregnant HIV positive woman on antiretroviral therapy, who was diagnosed with primary CMV-infection after 30 weeks gestation. She was treated with intravenous ganciclovir iv for a week and thereafter valacyclovir orally until the end of pregnancy. She was CMV-PCR negative after 4 weeks treatment, and delivered after 37 weeks’ gestation a small child (2.07 kg) in whom a prenatal CMV infection could be ruled out. The second case was a probable primary co- infection with HIV and CMV. A 25-year-old Caucasian male developed meningitis, hepatitis, myelopathy in the legs and retinal cotton wool spots at examination of the eyes but no CMV-retinitis. He was treated with combination antiretroviral therapy (c-ART) for the HIV-infection. During 10 weeks he developed IgG and IgM antibodies to CMV, HIV-RNA in the blood was 386 000 copies and CMV-PCR was 690 copies/ml. The CD4 count was 801 × 106/L. He improved upon continued c-ART, even if he did not receive CMV-treatment. However the authors conclude that the overlapping manifestations of acute HIV-1 and CMV makes it difficult to determine how each virus infection contributed to his condition. None of these primary CMV-infection cases were associated with lymphoma or other tumour development. That CMV might be involved in the development of PTLD has been suggested [5]. Epstein-Barr-virus (EBV) is related to several types of malignant lymphomas, especially in HIV-infected patients, and also to post-transplant lymph proliferative disorder (PTLD) [5, 6]. HHV-8 is linked to Kaposi’s sarcoma, Castleman’s disease and to some lymphomas [7]. One HHV-8 associated ALCL has been documented in a HIV-positive patient [8]. In this patient there was no sign of EBV in the tumour but EBV-reactivation and CMV infection was not investigated. A malignant lymphoma in an HIV-positive patient with EBV and HHV-8 co infection has recently been described by Crane et al. [9]. This patient died within 3 months of diagnosis of the lymphoma. An ALCL with very rapid progression has also been described by Mosunjac et al. [10]. The development of HHV-8 infection is not as well described as the infection with the other human herpes viruses. Hladik et al. [11] studied serocorversion time after blood transfusion and found that most HHV-8 negative patients serocorverted in three to ten weeks after transfusion with HHV-8 seropositive blood. Similar seroconvertion times was also found by Sergeric et al and Gentile at al in patients after Bone Marrow Transplantation [12, 13]. Two cases of fatal disseminated Kaposi’s sarcoma following HHV-8 primary infections has been described by Marcelin et al. [14]. They died at 5 and 6 months after transplantation after a disease course of 3 to 4 weeks. Abate et al. [15] recently demonstrated other human herpes viruses than EBV in eight patients with Burkitt lymphoma. They found four patients with CMV in the adjacent non-neoplastic tissue, three with HHV-8 and in one patient EBV, CMV, HHV-8 and HTLV-1 was found simultaneously. All viruses were detected by immunohistochemistry and they suggest that these viruses might be a contributing factor to lymphoma development.

Here we present an asymptomatic HIV-infected Caucasian homosexual male with a primary CMV-infection, HHV-8 infection and concomitant reactivation of EBV, who developed a malignant lymphoma leading to death within 3 weeks.

## Results

### Clinical report

A 34-year old HIV-infected man presented acutely due to a 2 days’ period of fatigue and fever. Two years earlier he had been diagnosed with HIV-infection as a result of screening for sexually transmitted infections (STI). At the time of HIV-diagnosis he was in good health but with slightly enlarged neck lymph nodes. He was seronegative for hepatitis A, C and CMV, but had IgG antibodies to EBV and was seropositive for hepatitis B surface antigen and e-antigen. Liver enzymes were normal. As he was asymptomatic with a CD4 cell count > 500 × 106/L, he was followed up clinically but without antiretroviral medication. One year later the CD4 count still was >500 × 106/L.

On acute admission, the patient had enlarged lymph nodes in the neck and axillae. There was no palpable hepatic or spleen enlargement, but he had a slight pain upon examination of the left side of the abdomen. Auscultation of the heart and lungs was normal. After hospitalization the fever and malaise continued and in three days he had a massive enlargement of lymph nodes on both sides of the neck and in the axillae. The spleen was now palpable five centimetres below the left arc of the ribs and the liver 1–2 cm below the right arc. An opportunistic infection was suspected already day one but diagnostic tests for sepsis, tuberculosis, other mycobacterial infections, and *Pneumocystis jiroveci (carinii)* pneumonia were negative. Chest X-ray showed no abnormalities. The CD4 cell count was 540 × 106/L and his plasma HIV-RNA level was 31 000 copies/mL. An enlarged liver and spleen (15,5 × 8 cm) were confirmed by computer tomography. The histopathology of a lymph node removed from the left side of the neck was interpreted as reactive hyperplasia without signs of lymphoma. Bone marrow aspirate and liver biopsy showed reactive inflammation without signs of lymphoma or other malignancies. There were no signs of infections with CMV, mycobacterium or fungi.

### Progress and CMV-testing

**Fig. 1:**
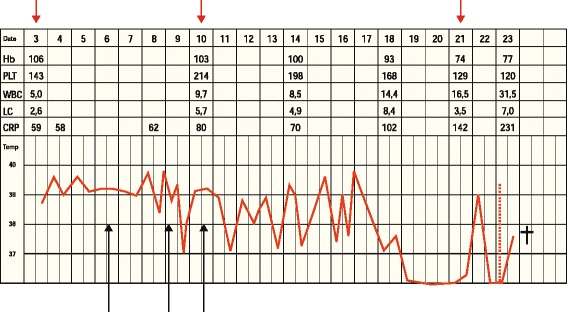
Overview of the in-patient history: temperature curve, blood cell parameters and Creactive protein. The continuous red line is the temperature curve. The red arrows above the curve indicate time points for CMV-, EBV- and HHV-8 serology and/or PCR. The long black arrows beneath show time points from left to right for lymph node biopsy, liver biopsy and bone marrow aspiration. HB = Haemoglobin in g/L, PLT = Platelet count x 1012/L, WBC = white blood cell count x 109/L, LC = lymphocyte count x 109/L, C-reactive protein in mg/L

The course of the disease with fever, blood parameters and date of biopsies are shown in Fig. 1. C-reactive protein (CRP) was moderately increased upon admission and did not change during the first 2 weeks. The patient had leucocytosis due to the increase of both neutrophils and lymphocytes and from day three until day 14 he had a pronounced lymphocytosis including activated lymphocytes microscopically resembling Downey- McKinley cells observed in patients with primary EBV- and CMV-infection. The majority of the lymphocytes (75 %) were T-cells. The CD4 cells increased from 550 × 106/L to 1240 × 106/L and the CD8s from 1390 × 106/L to 2970 × 106/L, while the ratio was unchanged (0.4) compared to the year before. CMV was identified by PCR and by a rapid isolation technique [16] of peripheral blood leucocytes on day three, providing a working diagnosis of a primary CMV-infection. Because of the fever a 3-day treatment with indomethacin was initiated on day 18, upon which the temperature decreased, but then reappeared on day 21.

Since the patient rapidly deteriorated with cerebral confusion, declining haemoglobin and platelets, treatment with foscarnet was initiated intravenously at 90 mg/kg twice daily on day 22. After 12 h the temperature normalised and the patient felt better. However, during the next 24 h the patient’s condition deteriorated and he died.

Autopsy showed Adult Respiratory Distress Syndrome (ARDS) and massive lymphoma infiltration in liver, spleen and multiple lymph nodes, which motivated a re-evaluation of the course of the disease and the potential role of herpes viruses in the development of the disease.

### Detection of CMV, EBV and HHV-8

Serum and plasma samples were analyzed for CMV IgG and IgM, EBV IgG and IgM, HHV-8 IFT (antibodies against lytic antigens) and HHV-8 LANA (late nuclear antigen) antibodies (Table 1), as well as CMV-DNA, EBV-DNA, HHV-8-DNA and HIV-RNA (Table 2).

**Table 1:** Serological analysis of CMV, EBV and HHV-8 antibodies over time

Test used	9 months prior to admission	Day 3	Day 10	Day 21
CMV IgG EIA	<100^a^	< 100^a^	500^b^	1000^b^
CMV-IgM capture EIA	<100^a^	< 100^a^	100^b^	Close to cut-off^a^
EBV-EIA (EBNA) IgG	> = 20^b^	> = 20^b^		> = 20^b^
EBV-IgG VCA EIA, U/mL	75^b^	120^b^		130^b^
EBV-IgM EIA	Negative	Negative		<= 40^a^
HHV-8 IFT (lytic antigens)	Negative	Positive		Positive
HHV-8 LANA (latent nuclear antigens)	Negative	Negative		Negative

*EIA* enzyme immunosorbent assay, *VCA* viral capsid antigen

^a^negative

^b^positive

**Table 2:** Detection of CMV, EBV, and HIV in blood samples over time

Test used	9 months prior^a^) to admission	Day 3^a^)	Day 10^b^)	Day 21^a^)
CMV-isolation, classical and rapid			Positive	
Quantitative CMV-DNA in plasma/serum (copies/mL)	Negative	3700	6000	2700
Quantitative EBV-DNA in plasma/serum (copies/mL)	Negative	250	520	5600
Quantitavie HHV-8-DNA In plasma/serum (copies/mL)	Negative	Negative	11000	54000
Quantitative HIV-RNA in plasma/serum (copies/mL)	30,000	31000		27000

^a^)tested in serum

^b^)tested on EDTA-blood

Furthermore, the samples were tested for antibodies and PCR for herpes simplex virus (HSV) 1, 2 as well as Varicella Zoster Virus (VZV) and HHV-6, -7. The patient, earlier seronegative, had increasing CMV-IgG and -IgM during the disease.

CMV-PCR increased as well during the acute disease. The patient had EBV antibodies before the acute disease which did not change significantly during the acute disease. EBV-DNA, negative the year before his acute disease, increased during the last 2 weeks. The patient was HHV-8 sero- and PCR negative the year before acute disease. He presented with a positive HHV-8 serostatus (antibodies to lytic antigen) at day three of the acute disease but the HHV-8 LANA antibodies were negative throughout the disease His HHV-8 DNA load however increased during his disease from undetectable on day three to 54000 copies/mL on day 21 (Tables 1 and 2). The HIV-RNA levels were unchanged compared to the previous year. HIV-1 was shown to be of subtype B and of the R5 co receptor use/phenotype. HSV 1 and 2, VZV, HHV-6 and HHV-7 measured with PCR in blood were negative throughout the disease. There were no change in IgG of the other herpes viruses investigated (HSV1-2, VZV, HHV-6 and 7.)

### Morphologic, immonohistochemical and flow cytometric analysis

**Fig. 2:**
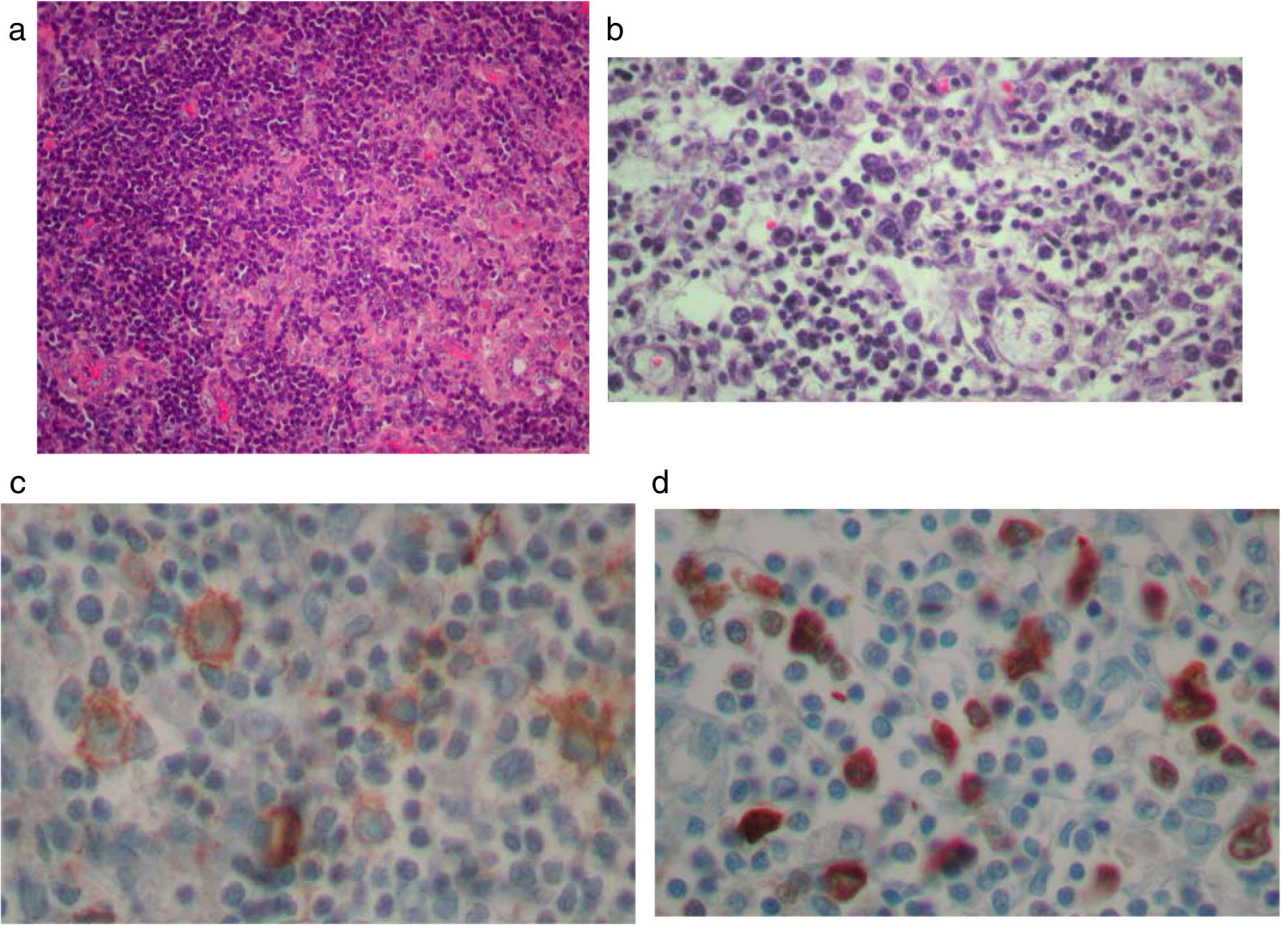
**a** Histopathology of a lymph node at autopsy, showing ALCL. The inset shows enlargement of tumour cells. **b** ALCL Magnification x 20. **c** CD30 immunohistochemestry positive. **d** Ki-67 immunohistochemestry positive

Tissue samples from lymph node, liver and bone marrow biopsies, and post mortem samples were stained with hematoxylin and eosin, according to standard protocols. (EBER) by an in situ hybridization technique (Dakopatts) was performed on biopsies and autopsy material. At autopsy the patient macroscopically had an enlarged liver and spleen. Enlarged lymph nodes were found in the mediastinum, the abdomen and adjacent to the liver. All other organs, including the brain, were normal. Microscopic examination showed that the lung alveoli were filled with fluid that can be observed in ARDS. The lymph nodes, bone marrow, and spleen showed infiltration by a large cell malignant lymphoma. The lymphoma at autopsy was analysed using a streptavidin-biotin procedure with monoclonal antibodies against the following cellular differentiation (CD) antigens: CD20, CD79e, CD2, CD3, CD4, CD5, CD7, CD8, CD30, CD15, and also the ALCL markers ALK-1 and Ki-67, showing immune profile consistent with CD30-positive ALK-1 negative ALCL Immunohistochemistry showed an immune profile consistent with CD30-positive ALK-1 negative ALCL (Table 3 and Fig. 2).

**Table 3:** Results of immunohistochemistry

	Lymph node biopsy	Lymphocytes at autopsy	Lymphoma cells at autopsy
H + E	Small focus ALCL		ALCL
CD20			negative
CD79a			negative
CD3 (T-cell)			negative
CD2			negative
CD7		positive	negative
CD68			negative
CD30	ALCL cells positive		positive
LMP1			negative
EBER		Some positive	negative
CD 57			negative
K-1			positive
Granzym B			A few cells positive
CD10			negative
CD8			negative
CD4			negative
BCL-2		positive	negative
CD56			negative
CD5		failed	failed
CD38		negative	positive
CD15		failed	failed
P53			A few positive cells
CD10		negative	negative
ALK-1			negative
Ki-67		negative	positive

Immunohistochemistry for CMV was negative. There were no spindle cells typical of Kaposi’s sarcoma in the lymphoma or adjacent tissue. There was no sign of haematophagocytic histiocytosis in the bone marrow.

## Discussion

This is the first report of a malignant lymphoma with signs of reactivation of EBV concomitant with both primary CMV-infection and HHV 8 infection in an HIV infected patient. The lymphoma was diagnosed as ALCL. To our knowledge this is the first report of a lethal primary CMV-infection in an HIV-infected patient with a well-preserved immune system. The HHV-8 infection was verified by seroconversion and increasing levels of HHV-8 PCR in blood and could be a contributing factor.

Autopsy showed that the immediate cause of death was ARDS, which is reported to occur during primary CMV-infection and in patients with severe malignancies [17, 18]. Contributing to the cause of death was the massive infiltration of ALCL cells in bone marrow, lymph nodes, and spleen. Before the acute illness there were no signs or symptoms of generalised lymphoma. Three weeks before onset of symptoms the patient had unprotected sex which could be possible source of his CMV and also HHV-8 infection. The absence of CMV IgM and IgG antibodies early during the acute illness in combination with the seroconversion after 1 week, support the diagnosis of a primary CMV infection. The detection of CMV by virus isolation, rapid isolation, and by PCR [19], concomitant with the detection of viral DNA in plasma confirmed that the patient experienced a primary CMV infection (Table 1). Furthermore, the significant increase in CD4 and CD8 lymphocytes is compatible with such a primary infection [20]. The seroconversion for HHV-8 and the rapid HHV 8-DNA increase is consistent with active ongoing infection. The patient was probably infected at the same time as when he was infected by CMV. The seroconversion time for HHV-8 is similar to the other herpes viruses [11]. The HHV-8 LANA antibodies was negative throughout his disease but this method is not as sensitive as the HHV-8 IFT test [14]. The earlier reports on primary CMV-infection in HIV-patients have not been linked with lymphoma nor with an aggressive and lethal course. Two previously reported cases had been treated with antiretroviral therapy [3, 4]. One was treated with gancyclovir for the CMV infection and responded with a rapid resolution of symptoms. The other had a protracted course, but the role of CMV could not be evaluated, because the symptoms could equally well be due to the concomitant primary HIV infection.

 In this case CMV-PCR in blood was only slightly elevated in contrast to our patient. These two patients were not analysed for EBV, HHV 8 or other herpes viruses.

Our patient’s latent EBV-infection was reactivated shown by the increasing EBV-DNA levels in serum [21] and EBER was detected in lymphocytes EBV is associated with many forms of malignant lymphoma in AIDS patients including the CNS-NH lymphomas which are 100 % EBV-associated [22]. While severe CMV-related CNS disease is often found in AIDS cases, CMV has rarely been found to be directly associated with CNS lymphomas, except in one study, where CMV inclusions were found in the cerebral neoplastic cells [23]. CMV is also connected to EBV-initiated post-transplant lymph proliferative disease (PTLD) in patients after transplantations with bone marrow or solid organs [5, 6] related to the immunosuppressive therapy. A direct relationship between a primary CMV-infection, EBV reactivation and ALCL or other lymph proliferative disorders, such as PTLD, has not been reported. However the presence of CMV together with HHV-8 in the EBV driven endemic Burkitt lymphoma recently observed indicate a possible role for other herpes viruses than EBV in the development of lymphomas. In a few cases of early primary effusion Lymphoma (PEL) the neoplastic cells have shown to be co infected with EBV and HHV-8 [24]. The ALCL is considered to be a T-Cell derived lymphoma but association with EBV or HHV-8 has only been reported in a few cases [7]. In the chronic stage HIV-infection can interfere with the immune system at many levels that will facilitate tumour development (oncogenesis) as well as CMV infection. CMV was not found in the lymphoma cells but can also facilitate tumour development by interfering with the immune system. Crane et al. [9] report on a co infection with EBV and HHV-8 in a vascular ALCL in an HIV positive patient with well preserved immune system. This patient got c-ART and had a CD4 count above 500. Co-infection with EBV and HH-8 in this patient was detected using situ hybridisation for EBER and HHV-8.

Our patient had evidence of five viral infections HIV, chronic HBV, latent EBV and primary CMV and HHV-8. All of these five viruses can have contributed to the development of the lymphoma. During the course of the disease we have seen a rapid increase in CMV-DNA, EBV-DNA and HHV-8 DNA in the blood simultaneously with the development of the lymphoma supporting the earlier findings, that both EBV and HHV-8 could be involved in the tumor development. However neither HIV-RNA levels, nor the CD4/CD8 ratio changed during the course of the disease, implying that the HIV-infection was not directly influenced by the reactivation of the EBV-infection or the development of the lymphoma. However, the HIV-infection might have caused an immunodeficiency that contributed to the tumour development. HBV has only been associated with hepatocellular cancer as an effect of the chronic inflammation in the liver. There is no increased risk of haematological malignancies in HIV-patients with chronic hepatitis B compared to other HIV-infected patients as shown by Jou et al. [25]. HHV-8 infects dendrite cells and B cells [26]. HHV-8 show a very particular protein profile in latency, that has not been identified in other herpes viruses, or viruses in general [27]. During active replication viral IL6, IRF-, IRF-3, K1 and K15 are expressed maximally but they are also detected subsequently in the latent cells. IL6 is a powerful inducer of inflammation and might affect EBV- reactivation, HHV-8 infection or primary CMV infection and even contribute to oncogenic process. The complex interactions of HIV, CMV and HHV-8- and even HBV could have provided the necessary combination of immune suppression and immune stimulation for the lymphoma genesis to precede and accelerate, even though the HIV-infection was not advanced, as measured by HIV-RNA and CD4 counts. We found no primary infection or reactivation of the other human herpes viruses investigated (HSV-1 and 2, VZV, HHV-6 and 7) in this patient. A rapid course (weeks to a few months) of ALCL in a subset of patients has been reported [10]. However, in these patients the development and role of CMV, EBV and HHV8 was not investigated. It would have been interesting to know if rapid lymphoma progression in these patients was correlated to reactivation of CMV, EBV, HHV8 or other viruses. Furthermore, the increase in EBV-DNA, HHV8-DNA in blood that was found in our patient during the cause of the disease might be used as an indicator of the severity of the lymphoma that is under development in a patient with an unclear lymphoproliferative disorder and might also be of help when evaluating the therapeutic response for this lymphoma as has been suggested earlier [28]. It is relevant to look for more than one virus having a role in lymphoma development with both serology and PCR-techniques, especially if you have an unusually rapid progression of the lymphoma. If CMV-PCR increases as a sign of developing CMV-infection treatment with gancyclovir should be started immediately to prevent further deterioration caused by CMV-infection. If both CMV and HHV-8 DNA are discovered foscarnet could also be used. There is a need for optimal and early anti-CMV treatment in patients with HIV infection and a primary CMV infection as well as HHV-8 treatment if HHV-8 infection is found. Today there is no good viral prophylaxis against CMV or HHV-8 infection but there is hope of a CMV-vaccine in the future. This case story is also an example of the hazard of having unprotected sex without condoms. The risk is for all HIV-infected patients, especially if the patient is CMV-seronegative.

Another issue is the need for specially designed cytostatic therapy for these lymphomas that seem to have a very poor prognosis compared to others. In our index patient the CMV treatment was introduced rather late and this might have been suboptimal to the outcome. Limitations of this study is that we have not investigated HBV in the lymphoma and that in situ hybritization for HHV 8 in the histology of the lymphoma failed.

## Conclusions

The rapid lymphoma development in our patient, together with the kinetics of the laboratory and pathological findings over a short period of time provide a conspicuous link between the three herpes virus infections and the tumour development. It is relevant to monitor patients for CMV, EBV and HHV-8 DNA in patients with rapidly progressing lymphoma to optimize therapy. It is also a reminder of the importance of prevention and prophylaxis of several infections by having protected sex.

## Methods

Analysing (or reading) patient record, Serology test for human herpesvirus 1-8, PCR tests for human herpes virus 1-8 on serum and/or plasma samples hematoxylin staioing and immunohistochemichal staining on lymphnode, bone marrow, liver biopsy as well as autopsy material.

## Abbreviations

ALCL, anaplastic large cell lymphoma; CMV, cytomegalovirus; EBV, epstein-barr virus; HIV, human immune deficiency virus; HHV-6, human herpes virus 6; HHV-7, human herpes virus 7; HHV-8, human herpes virus 8; HSV, herpes simplex virus; VZV, varicella zooster virus
